# Neurectomy for Facial Neuralgia, with Recovery

**Published:** 1889-10

**Authors:** E. M. Magruder

**Affiliations:** Charlottesville, Va.


ARTICLE II.

### NEURECTOMY FOR FACIAL NEURALGIA, WITH RECOVERY.

BY DR. E. M. MAGRUDER, OF CHARLOTTESVILLE, VA.

My first case was a gentleman, seventy-one years of age, who had had persistent neuralgia for fourteen years, that no medicine cured, although he had consulted eminent specialists. Seven drops of fluid extract of gelsemium, every three hours, gave greater relief than any other medicine except morphia. But all medicines failing, the patient was chloroformed, and, with strict antisepsis, an incision was begun just below the lower orbital margin, over the infra-orbital foramen, straight downward, parallel with the nose, toward the lip, and ended on a level with the lower border of the ala nasi—about an inch incision. The fascia and fibres of the levator labii superioris were torn through with the handle of the scalpel, and the nerve exposed at its exit, where it divided into two branches. Each branch—the palpebral, the nasal, and the labial—spreading into a fan-shape as it neared its destination, was dissected out as far as it could be followed without mutilating the face too much. The main trunk was then seized with forceps at the foramen, drawn out as far as possible without breaking it, and cut off close to the bone, after which the various branches were divided at their farthest point of dissection. The wound was closed with fine silk sutures. There was at first considerable paralysis of the side of the face, and loss of sensation, but these disappeared, except from the right half of the upper lip, which is still without motion or sensation. There has been no recurrence of the neuralgia. The patient feels like a new man.

Case II. was a lady, fifty-eight years of age, who has had facial neuralgia for ten years. At first it was confined to the left lower jaw, never passing the middle line of the chin; but afterward extended to the left external ear, temple, and side of head above and behind the ear (auriculo-temporal), to the left side of the tongue (gustatory), and then to the left side of the floor of the mouth (mylo-hyoid). The diagnosis was neuralgia of the inferior dental nerve, with reflex and sympathetic phenomena exhibited by the auriculo-temporal, gustatory, and mylo-hyoid nerves. As to treatment, teeth had been extracted, analgesics had been used, etc., and, finally, total neurectomy of the inferior dental nerve—including its branches—was done, with cure. In the operation, avoid injury of the facial artery and Steno's duct. Entering the scalpel just in front of the posterior border of the ramus, just below the parotid duct and lobe of the ear, a curvi-linear incision was made downward, half an inch in front of the inferior maxillary angle, then forward a little above the lower border of the ramus, and upward just behind, and avoiding the facial artery, stopping short of the line of Steno's duct above. The flap thus shaped was raised by shaving the masseter muscle from its attachment to the outer surface of the ramus, and the bone laid bare. With a half inch trephine cut out a button of bone from the centre of the outer plate of the ramus, exposing the nerve in its bony canal. Seizing the proximal end of the nerve with forceps, strong traction was made from the direction of its origin; it was then cut off with scissors, close to the bone, as it entered the circular cavity made by the trephine. Then, the wound being stuffed with moist antiseptic cotton, and the hæmorrhage stopped with pressure, a second incision was made, an inch long, horizontal in direction, over the mental foramen (below the root of the second lower bicuspid tooth), beneath the depressor anguli oris, disclosing the mental nerve and its branches beneath the last-named muscle. The nerve was grasped with forceps and pulled upon, but broke off at its point of exit. The branches were then dissected out and cut off as far as possible from the foramen. Then returning to the first wound over the ramus, and chiselling away the wall of the dental canal, or one-eighth inch from the circular cavity in the ramus, so as to expose this end of the nerve which had been divided by the distal side of the trephine, it was drawn out of the dental canal with forceps, in its entire length from the ramus to the foramen. In all, three and three-eighths inches of nerve-structure were removed. The wounds were closed, and the patient was perfectly relieved, without any return of neuralgia since. The paralysis of the left side of the face disappeared in about two weeks.

The lesson learned is, in all operations for facial neuralgia remove as much of the troublesome nerve and its branches as the anatomical formation of the parts will possibly allow without rendering the procedure too grave.—*Medical Record.*

---

ARTICLE III.

